# Reaching Heaven

**Published:** 1906-06

**Authors:** 


Reaching Heaven.

"Heaven is not reached by a single bound,
But we build the ladder by which we rise
From the lowly earth to the vaulted skies,
And we mount to its summit round by round."

*Exchange.*

